# Carbon Sequestration by Perennial Energy Crops: Is the Jury Still Out?

**DOI:** 10.1007/s12155-014-9571-0

**Published:** 2015-01-15

**Authors:** Francesco Agostini, Andrew S. Gregory, Goetz M. Richter

**Affiliations:** Department of Sustainable Soils and Grassland Systems, Rothamsted Research, Harpenden, Hertfordshire, AL5 2JQ UK

**Keywords:** Soil organic carbon, Switchgrass, *Miscanthus*, Model, Willow, Poplar, Short-rotation woody crops

## Abstract

Soil organic carbon (SOC) changes associated with land conversion to energy crops are central to the debate on bioenergy and their potential carbon neutrality. Here, the experimental evidence on SOC under perennial energy crops (PECs) is synthesised to parameterise a whole systems model and to identify uncertainties and knowledge gaps determining PECs being a sink or source of greenhouse gas (GHG). For *Miscanthus* and willow (*Salix* spp.) and their analogues (switchgrass, poplar), we examine carbon (C) allocation to above- and belowground residue inputs, turnover rates and retention in the soil. A meta-analysis showed that studies on dry matter partitioning and C inputs to soils are plentiful, whilst data on turnover are rare and rely on few isotopic C tracer studies. Comprehensive studies on SOC dynamics and GHG emissions under PECs are limited and subsoil processes and C losses through leaching remain unknown. Data showed dynamic changes of gross C inputs and SOC stocks depending on stand age. C inputs and turnover can now be specifically parameterised in whole PEC system models, whilst dependencies on soil texture, moisture and temperature remain empirical. In conclusion, the annual net SOC storage change exceeds the minimum mitigation requirement (0.25 Mg C ha^−1^ year^−1^) under herbaceous and woody perennials by far (1.14 to 1.88 and 0.63 to 0.72 Mg C ha^−1^ year^−1^, respectively). However, long-term time series of field data are needed to verify sustainable SOC enrichment, as the physical and chemical stabilities of SOC pools remain uncertain, although they are essential in defining the sustainability of C sequestration (half-life >25 years).

## Introduction

The term ‘carbon (C) sequestration’ describes processes by which atmospheric carbon dioxide (CO_2_) is captured and stored in a long-term reservoir. This review focuses on the role of green plants as principal agents of biologically captured and stored soil organic C (SOC). In particular, we collated evidence for dedicated perennial energy crops (PECs) to sequester C and quantitative data to parameterise SOC turnover models.

PECs are fast-growing (perennial) species that can be grown on marginal soils with low inputs [1, 2] to generate energy either from direct combustion or via conversion to liquid fuels. The area of land devoted to PECs is likely to increase as countries seek to reduce their dependence on fossil fuels and greenhouse gas (GHG) emissions for climate change mitigation. This will require land conversion to energy crops, and questions over how much land is available and where PECs should be grown are debated at present amongst both scientists and policymakers [3]. Pertinent to these is a better understanding of SOC changes associated with different cropping systems, and the quantity and the quality of the residues returned to the soil [4].

PECs are considered to be a nearly C-neutral source of energy [4] as, on average, for each 0.6 kg of fossil fuel C used in cultivation, 1 kg of C is produced as biomass [5]. This estimate, however, can vary greatly according to the value chain and methodology employed, e.g. due to the selected boundary of the life cycle assessment (LCA), to differences in resource use efficiencies or soil and climate characteristics. In addition, PECs mitigate C emissions by their ability to sequester C from litter, harvest residues and roots into SOC. Although principal differences between PECs in terms of residue returns are known, no conclusions on the impact of harvest on SOC were drawn [5, 6]. It has been proposed that C sequestration under PECs should be at least 0.25 Mg C ha^−1^ year^−1^ in order to make the crop C-neutral when converted to biofuel [7]. To date, estimates of C sequestered under PECs range between 0.6 and 3.0 Mg C ha^−1^ year^−1^ [1, 5]. However, little experimental evidence is available to describe C sequestration after land use change (LUC) to PECs, where in the soil and when SOC will reach its equilibrium and how long it will last if PECs are replaced by other crops.

The critical components that affect C sequestration rate are inputs and turnover rates, but data on these are very limited in PEC systems. The impact of LUC to PECs on SOC dynamics needs to be quantified in terms of changes immediately after crop establishment (resilience phase) and its equilibrium state [8]. Although there have been previous reviews on C sequestration under PECs [8–11], some of these were either unrelated to SOC [12, 13], or did not report any experimental evidence [1, 14, 15], or focused on specific geographical regions [16].

The overall aim of this review was to evaluate the currently available quantitative experimental evidence of C fluxes in low-input PEC systems specific for modelling the transition from arable land. As changes in soils require long-term evidence, we focus on the needs in order to develop and parameterise improved SOC models to simulate such transient perennial systems. Specific focus for PEC systems is given to (1) defining the system components relevant to C sequestration; (2) collating and synthesising the experimental evidence of C input, turnover and sequestration rates; (3) outlining the modelling tools that simulate SOC dynamics; and (4) identifying the most important uncertainties for the quantification its SOC changes. In contrast to other analyses, we did not consider feedstock production with high nitrogen input, e.g. forage switchgrass [8], but focus on low-input ligno-cellulosic systems [17].

**Fig. 1:**
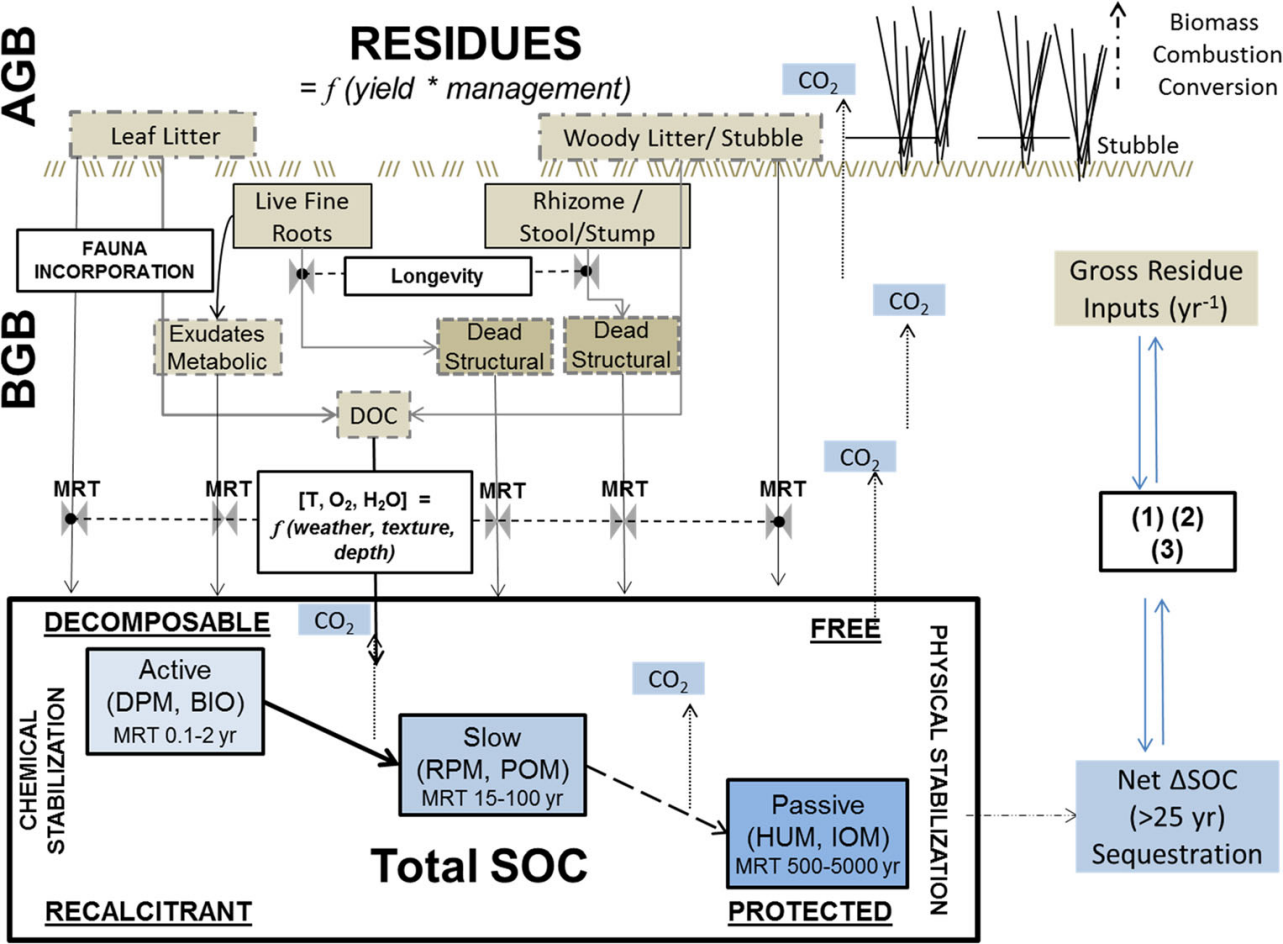
Conceptual model and components of carbon (*C*) dynamics under perennial energy crops (*PECs*): soil C pool/continuum; C source pools representing residues dispersed into the soil and their mean residence time (*MRT*), linked process-based models (*1*) soil–plant water dynamic, (*2*) soil biomass dynamic and (*3*) N limitation effect

Emphasis has been given here to synthesise current knowledge of the whole system (Fig. 1) and to fill inevitable gaps of evidence for ligno-cellulosic feedstocks from herbaceous and woody crops to improve our understanding of the long-term effects on SOC and to highlight where further data are required.

## Components of Perennial Energy Cropping (PEC) Systems

In the following sections, we first lay out the conceptual framework of the two main PEC systems we considered and then define the principal elements affecting their capability to sequester C into the soil (choice of crops, management, environmental control factors and yield) as these formed the basis of the evidence search outlined. Focus was given to *Miscanthus* and willow but, due to the scarcity of some direct experimental evidence in these crops, data from two proxy crops, switchgrass and poplar, have also been used.

### PEC Systems

PEC systems consist of herbaceous crops and grasses (HCG) or short-rotation woody crops (SRWC), managed as short-rotation coppice (SRC) or short-rotation forestry (SRF). Worldwide, the main HCGs are C_4_ grasses *Miscanthus* (*Miscanthus* spp. L.) and switchgrass (*Panicum virgatum* L.) and C_3_ grasses such as reed canary grass (*P* *halaris arundinacea* L.) and giant reed (*Arundo donax* L.). The main species planted as SRC are willow (*Salix* spp. L.) and poplar (*Populus* spp. L.), both being fast-growing. Depending on climate and soil conditions, different species are used for SRF [2, 18]. *Miscanthus* and switchgrass provide greater yields in warm and temperate regions than any other grasses [2, 18] in spite of *Miscanthus* showing higher sensitivity to drought. Reed canary grass and giant reed seem better suited to cooler northern Europe and Mediterranean regions, respectively.

Perennial systems are characterised by long-term occupation of land, continuous biomass production with variable harvest cycles (1–15-year duration), continual residue addition to the soil and little disturbance of soil and belowground biomass (BGB). The management of such systems greatly affects residue inputs as harvest practices vary the amount of aboveground biomass (AGB) removed depending on harvest date [19, 20] and harvest method [21, 22]. Eventually, the reversion of the perennials to arable will destroy the BGB in the plough layer, which could be removed or retained, decomposed and humified to become part of SOC (Fig. 1).

*Miscanthus*, switchgrass, willow and poplar may increase long-term SOC pools due to their extensive root systems [1]. LCA on data from the USA found poplar and switchgrass provided the largest overall GHG sinks [18], with poplar superior due to the fact that in grass systems, nearly all AGB is removed. *Miscanthus* seems to be the best choice in terms of C sequestration and input efficiency [23] due to its slow decay of residues and high BGB [13]. If *Miscanthus* is the best option for temperate Europe, switchgrass could be a better choice for dry areas and relatively poor soil quality [2]. The main input of C from switchgrass to SOC comes from its dense root system in the top 30 cm [24], although its residues have a fast C turnover [25].

SRWC PEC systems can increase the C stored belowground for a relatively long time, with contributions from dead wood [16]. Clearance at termination could leave variable amounts of BGB as coarse root and stumps [26]. Willow and poplar are favoured for SRC due to their high growth rates and broad genetic variability, which allows adaptation to different soil and climate conditions [27, 28]. It is likely that the amount of litter deposition is influenced by yield potential [8, 10, 29].

### Components of the C Cycle Under PEC Systems

The C cycle of PECs is assumed to be divided into qualitatively different inputs from AGB and BGB (Fig. 1). Whilst harvested AGB will be converted to atmospheric CO_2_ by combustion, litter and harvest residues undergo a phase of surface decomposition and incorporation. BGB components will enter the SOC pools dependent on their mortality/longevity [30–32]. Residue quantity, quality, decomposition rates and, hence, SOC dynamics differ between HCG and SRWC systems [33], and this review attempts to collate evidence for the annual gross residue inputs. Residues decompose according to a decay rate (*k*), dependent on composition, soil protective characteristics, microbial biomass and environmental conditions (temperature, precipitation, soil texture and water availability), which determine the system and compound specific mean residence time (MRT) [34, 35]. The amount of C transferred to soil is a function of several system components [10] such as (i) litter and harvest residues and their decomposition rates; (ii) BGB, its longevity, composition and decomposition rates; and (iii) depth and distribution of the root system. Therefore, each PEC system will affect SOC both directly through the composition and mass of plant residues and indirectly through its impact on the soil environment [36, 37].

Modelling SOC dynamics under PECs requires knowledge of the above-mentioned soil components, ideally integrated with simulated C capture and allocation to crop biomass components to the soil [38, 39]). In view of the main components defined here, our conceptual framework (Fig. 1) aims to align gross input components with SOC fractions of simulation models (see below; [34]). Environmental variables, such as soil water dynamics [40], temperature [38, 41], litter incorporation and dissolved organic C (DOC) transport [42] and nitrogen [14, 43], affect C cycling under perennial vegetation.

### Review Method: Meta-Analysis and Data Synthesis

**Fig. 2:**
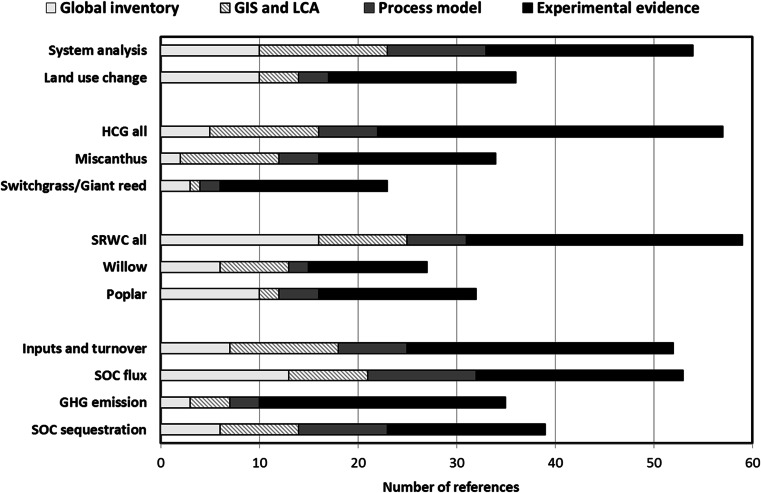
Bar graph describing the meta-analysis of the papers on herbaceous crop/grass (*HCG*) and short-rotation woody crop (*SRWC*) perennial energy crop systems selected and reviewed for the present work. Each paper was assigned to one or more of 12 classes defining its main objective and then further allocated into one of four classes defining the main methodology used

The search for published evidence on the components of the C cycle under PECs was carried out using the web-based search engines ISI Web of Knowledge^SM^ (Thomson Reuter, New York, USA) and Google Scholar (Google, CA, USA). We initially used the keywords ‘Biomass crop OR Energy crop OR Perennial crop combined with ‘GHG’, ‘Soil C’, ‘Modelling’, ‘Residues’, ‘Litter’, ‘Root’, ‘Decomposition’, ‘Turn over’ and ‘DOC’ covering the time period from 1994 to 2014. Due to the abundance of papers, we refined the search using key genera for bioenergy crops, omitting GHG balance and focusing on C sequestration. We further refined the search by searching for specific perennials, like *Miscanthus*, *Salix*/willow, *Panicum*/switchgrass and *Populus*/poplar as well as *A. donax*/giant reed. The literature was organised according to its methodology and scope in four groups: global inventory, geographic information system (GIS) modelling and/or LCA; process-based modelling; and experimental evidence. Twelve topical groups were distinguished according to the information they could provide in terms of the overall objective (Fig. 2).

Initially, the largest number of papers dealt with assessing GHG emissions or offsets based on LCA or GIS modelling, which carry the main uncertainty that underlying C sequestration models were not specifically calibrated for PECs [44]. Another large subset of papers took a global perspective extrapolating field trials using models assuming a generalised management. Experimental evidence was reported in 85 papers, of which 44 were specific field trials for the PECs under consideration and a total of 57 studies had data useful for parameterising C sequestration (Tables 1, 2 and 3). Although some papers recorded specific fractions of SOC [11, 45, 46], little information was available on parameters affecting SOC accumulation in slowly decomposable or ‘recalcitrant’ forms [25, 35, 47]. A considerable number of papers using process models were published (47), but few studies modelled C specifically under *Miscanthus* (nine) and willow (six). Some parameter gaps could be filled using data in papers on switchgrass (four) and poplar (four), but it is questionable whether parameters from other woody systems (e.g. evergreen forest) were appropriate for this purpose. Experimental evidence for directly measured GHG emissions was found in 12 papers from eight different experiments, mainly on nitrous oxide (N_2_O) from *Miscanthus* [48–51], switchgrass [52, 53], SRC willow and/or SRC poplar, mainly following arable [48, 49, 54] but some also after grassland [55, 56]. Some papers cover other GHGs, like CO_2_ and/or methane [48, 49, 53–55, 57].

**Table 1:** Gross carbon input to soil under herbaceous crop/grass and short-rotation woody crop systems from different sources under different environmental conditions. The specific crop systems are *Miscanthus* (principally *M*. × *giganteus*), switchgrass and giant reed for HCG and willow and poplar for SRWC. Data not available or undisclosed is indicated (n/a)

System	Crop	Source	Age (year)	Depth (cm)	Soil texture	Conditions (MAT; MAP^a^)	Method	Input (Mg C ha^−1^ year^−1^)	Reference	
HCG	M	Leaf	2	Surface	Silt loam	10.7 °C; 630 mm	C analyser	1.39	Amougou [69]	
		Leaf	3	Surface	Silt loam	11 °C; 713 mm	C analyser	1.50	Amougou [64]	
		Litter	2–4	Surface	Silty clay loam	9.9 °C; 652 mm	Biomass (43 % C)	0.89	Christian [65]	
		Litter	4–8	Surface	Silt clay loam	8.9 °C; 589 mm	TOC analyser	3.20	Beuch [62]	
		Litter	14	Surface	Sandy	9.3 °C; 830 mm	Mass spectrometry	3.11	Dondini [135]	
		Litter	3.5	Surface	Clayey silt	11.0 °C; 1042 mm	CHNSO analyser	2.17	Anderson-Teixeira [57]	
		Rhizome	2–4	0–23	Silty clay loam	9.9 °C; 652 mm	Biomass (43 % C)	1.17	Christian [65]	
		Rhizome^b^	3	0–30	Silt loam	11 °C; 713 mm	C analyser	2.66	Amougou [64]	
		Rhizome	4	0–30	Sandy	9.3 °C; 715 mm	Biomass (43 % C)	1.70	Himken [85]	
		Rhizome	6–8	0–40	Silt clay loam	8.9 °C; 589 mm	Biomass (43 % C)	1.16	Beuch [62]	
		Rhizome	7	0–25	Fine silty	11.1 °C; 1023 mm	C analyser	1.40	Dohleman [66]	
		Rhizome	14	0–35	Silty clay loam	9.3 °C; 704 mm	C analyser	1.01	Richter [80]	
		Root	2–4	0–23	Silty clay loam	9.9 °C; 652 mm	Biomass (43 % C)	0.19	Christian [65]	
		Root	3	0–30	Silt loam	11 °C; 713 mm	C analyser	0.25	Amougou [64]	
		Root	5	0–120	Fine silty	10–40 % SWC^c^	Root scan	0.68	Monti [61]	
		Root	5–6	0–180	Sandy loam	9.3 °C; 715 mm	Biomass (43 % C)	0.86	Neukirchen [84]	
		Root	6–8	0–40	Silt clay loam	8.9 °C; 589 mm	Biomass (43 % C)	0.38	Beuch [62]	
		Root	7	0–100	Fine silty	11.1 °C; 1023 mm	C analyser	0.55	Dohleman [66]	
		Root	14	0–100	Silty clay loam	9.3 °C; 704 mm	Biomass (43 % C)	0.42	Richter [80]	
		BGB^d^	14	0–60	Sandy	9.3 °C; 830 mm	Mass spectrometry	2.93	Dondini [135]	
		BGB^d^	3.5	0–100	Clayey silt	11.0 °C; 1042 mm	CHNSO analyser	1.09	Anderson-Teixeira [57]	
		All residue	9	0–100	Loamy sand	7.4 °C; 706 mm	Mass spectrometry	3.00	Hansen [106]	
		All residue	16					3.94		
	SG	Litter	3	Surface	Sandy loam	n/a; 404 mm	C analyser	1.50	Frank [67]	
		Litter	3.5	Surface	Clayey silt	11.0 °C; 1042 mm	CHNSO analyser	2.02	Anderson-Teixeira [57]	
		Litter	4	Surface	Silt loam	16.0 °C; 1180 mm	CN analyser	1.04	Garten [71]	
		Litter	5	Surface	Silty clay loam	n/a; 723 mm	Biomass (40 % C)	2.17	Wienhold [77]	
		Litter	6	Surface	Loam	n/a; n/a	Biomass (40 % C)	2.57	Tufekcioglu [72]	
		Crown	3	Surface	Sandy loam	n/a; 404 mm	C analyser	3.38	Frank [67]	
		Rhizome	7	0–25	Fine silty	11.1 °C; 1023 mm	C analyser	0.47	Dohleman [66]	
		Rhizome	4	0–30	Silt loam	16.0 °C; 1,180 mm	CN analyser	0.89	Garten [71]	
		root	4	0–90	Silt loam	16.0 °C; 1,180 mm	CN analyser	1.32	Garten [71]	
		Root	3	0–110	Sandy loam	n/a; 404 mm	C analyser	1.80	Frank [67]	
		Root	5	0–120	Fine silty	n/a; 10–40 % SWC^c^	Root scan	0.77	Monti [61]	
		Root	7	0–100	Fine silty	11.1 °C; 1,023 mm	C analyser	0.65	Dohleman [66]	
		Root	4	75	Sandy loam	n/a (AL; USA)	n/a	0.81	Bransby, [9]	
		Root	3	90	Fine sand	10.5 C; 178 mm	CNS-2000, IRMS	1.29	Collins [47]	
		Fine root	6	0–35	Loamy	n/a; n/a	Biomass (40 % C)	0.65	Tufekcioglu [72]	
		BGB^d^	3.5	0–30	Clayey silt	11.0 °C; 1,042 mm	CHNSO analyser	1.21	Anderson-Teixeira [57]	
		BGB^d^	4	75	Sandy loam	n/a (AL; USA)	n/a	1.63	Bransby [9]	
	GR	Root	5	0–120	Fine silty	10–40 % SWC^c^	Root scan	1.21	Monti [61]	
SRWC	W	Leaf	4	Surface	Silt loam	9 °C; 981 mm	C analyser	1.16	Pacaldo [131]	
			19					1.90		
		Litter	3	Surface	Clay	5.8 °C; 544 mm	Biomass (43 % C)	0.96	Rytter [58]	
		Litter	3	Surface	Sand	5.8 °C; 544 mm	Biomass (43 % C)	0.63	Rytter [58]	
		Coarse root	4	Surface	Silt loam	9 °C; 981 mm	C analyser	0.34	Pacaldo [131]	
			19					0.14		
		Fine root^e^	4	Surface	Silt loam	9 °C; 981 mm	C analyser	0.63	Pacaldo [131]	
			19					0.17		
		Fine root^e^	3	0–90	Clay	5.8 °C; 544 mm	Biomass (43 % C)	2.35	Rytter [58]	
		Fine root^e^	3	0–90	Sand	5.8 °C; 544 mm	Biomass (43 % C)	1.15	Rytter, [58]	
		Fine root	2–3	0–70	Sand (washed)	5.6 °C; n/a	Biomass (43 % C)	2.19	Rytter [82]	
	P	Leaf	5	Surface	n/a	15 °C; 676 mm	Biomass (43 % C)	2.48	Cotrufo [78]	
		Leaf	4–10	Surface	Clay loam	14.3 °C; 964 mm	Dry combustion	1.98	Fang [79]	
		Litter	2–4	Surface	Clay loam	3 °C; 463 mm	TOC analyser	0.61	Arevalo [60]	
		Litter	3	Surface	Loam	16 °C; 735 mm	Isotopes	2.67	Gielen [99]	
		Litter	9–11	Surface	Clay loam	3 °C; 463 mm	TOC analyser	4.68	Arevalo [60]	
		Litter	6	Surface	Loam	n/a; n/a	Biomass (40 % C)	1.13	Tufekcioglu [72]	
		Stump	3	Surface	Loam	16 °C; 735 mm	Isotopes	0.42	Gielen [99]	
		Root	4–10	n/a	Clay loam	14.3 °C; 964 mm	Dry combustion	1.17	Fang [79]	
		Coarse root	3	0–40	Loam	16 °C; 735 mm	Isotopes	0.66	Gielen [99]	
		Coarse root	2–4	0–30	Clay loam	3 °C; 463 mm	TOC analyser	0.22	Arevalo [60]	
		Coarse root	2–8	0–40	Sandy loam	10 °C; 417 mm	Biomass (43 % C)	0.32	Zhang [89]	
		Coarse root	9–11	0–30	Clay loam	3 °C; 463 mm	TOC analyser	1.45	Arevalo [60]	
		Coarse root	2–12	0–40	Sandy loam	8.4 °C; 204 mm	Biomass (43 % C)	0.16	Yan [93]	
		Coarse root	3	0–70	Silt loam	n/a; n/a	Biomass (43 % C)	0.78	Calfapietra [90]	
		Fine root^e^	2–4	0–30	Clay loam	3 °C; 463 mm	TOC analyser	0.46	Arevalo [60]	
		Fine root^e^	3	0–40	Loam	16 °C; 735 mm	Isotopes	0.94	Gielen [99]	
		Fine root^e^	2–8	0–40	Sandy loam	10 °C; 417 mm	Biomass (43 % C)	1.28	Zhang [89]	
		Fine root^e^	9–11	0–30	Clay loam	3 °C; 463 mm	TOC analyser	0.83	Arevalo [60]	
		Fine root	2	0–15 cm	Sandy	9.5 °C; 726 mm	Soil coring	0.17	Berhongaray [81]	
		Fine root	3–15	0–40	Sandy loam	8.4 °C; 204 mm	Biomass (43 % C)	0.03	Yan [187]	
		Fine root	7	0–5	Silt loam	n/a; n/a	Biomass (43 % C)	0.02	Abou-Jaoude [152]	
		Fine root	6	0–35	Loamy	n/a; n/a	Biomass (43 % C)	0.46	Tufekcioglu [91]	
		Fine root	19	0–150	Clay	10.4 °C; 630 mm	Dry combustion	0.06	Upson [92]	

*C* carbon, *HCG* herbaceous crops/grasses, *SRWC* short-rotation woody crop, *M Miscanthus*, *SG* switchgrass, *GR* giant reed, *W* willow, *P* poplar, *n/a* not available, *MAT* mean annual temperature, *MAP* mean annual precipitation

^a^Mean annual temperature and mean annual precipitation, unless stated otherwise

^b^Two to 13 % necrotic

^c^Soil water content (%)

^d^Belowground biomass

^e^Fine roots turnover up to six times annually [32, 58, 83]; we present snapshot values

**Table 2:** Mean residence time of different sources of herbaceous crops/grasses and short-rotation woody crop systems under different environmental or controlled conditions. The specific crop systems are *Miscanthus* (principally *M*. × *giganteus*) and switchgrass for HCG and willow and poplar for SRWC. Data not available or undisclosed is indicated (n/a)

System	Crop	Source	Age (year)	Depth	Soil texture	Conditions^a^	Method	MRT (year)	Reference
HCG	M	Leaf	4–6	Surface	Loamy sand	25 °C; 12 % SWC	Incubation	0.90	Beuch [62]
		Leaf and stem	n/a	Surface	Silt loam	13 °C; 1,444 mm	Litter bag	1.28–1.39^b^	Kim [122]
		Leaf and stem	n/a	Surface	Sandy loam	3 °C; 45 % WFPS	Litter bag	3.19	Magid [108]
		Leaf and stem	n/a	Surface	Sandy loam	9 °C; 45 % WFPS	Litter bag	1.09	Magid [108]
		Stubble	4–6	Surface	Loamy sand	25 °C; 12 % SWC	Incubation	0.63	Beuch [62]
		Litter	2	Surface	Silt loam	15 °C; −80 kPa	Incubation	1.37	Amougou [64]
		Litter	1–3	Surface	Silt loam	10.7 °C; 630 mm	Litter bag	1.85	Amougou [69]
		Litter	n/a	Surface	Sandy loam	15 °C; 20 % SWC	Incubation	0.11–0.12^c^	Ernst [110]
		Litter	n/a	Surface	n/a	n/a	Litter bag	1.96	Yamane [123]
		Rhizome	2–3	Surface	Silt loam	15 °C; −80 kPa	Incubation	1.20–1.40	Amougou [64]
		Rhizome	4–6	Surface	Loamy sand	25 °C; 12 % SWC	Incubation	0.66	Beuch [62]
		Root	2–3	Surface	Silt loam	15 °C; −80 kPa	Incubation	2.40	Amougou, [64]
		Root	4–6	Surface	Loamy sand	25 °C; 12 % SWC	Incubation	1.18	Beuch [62]
	SG	Leaf	2	Surface	Fine loamy	25 °C; 60 % WFPS	Incubation	3.13^b^	Johnson [4]
		Litter	n/a	Surface	n/a	10–16 °C; n/a	Modelled	0.85^d^	Garten, [8]
		Stem	2	Surface	Fine loamy	25 °C; 60 % WFPS	Incubation	3.16^b^	Johnson [4]
		Root	2	Surface	Fine loamy	25 °C; 60 % WFPS	Incubation	3.31^b^	Johnson [4]
		Coarse root	n/a	0–30 cm	n/a	10–16 °C; n/a	Modelled	1.50^e^	Garten, [8]
		Fine root	n/a	0–30 cm	n/a	10–16 °C; n/a	Modelled	0.75^d^	Garten, [8]
SRWC	W	Leaf	1	Surface	Clay	5.5 °C; 660 mm	Litter bag	2.80^f^	Slapokas [111]
		Leaf	1	Surface	Clay	5.5 °C; 660 mm	Litter bag	1.20^g^	Slapokas [111]
		Fine root	2	0–10 cm	Sandy loam	10.7 °C; 800 mm	Litter bag	3.70–7.14^b,h^	Püttsepp [127]
		Fine root	1–5	0–50 cm	Clay	5.8 °C; 544 mm	Rhizotron	0.14–0.25	Rytter [83]
		Fine root	3	0–90 cm	Clay and sand	5.8 °C; 544 mm	Lysimeter	0.15–0.16	Rytter [58]
		Fine root	4	0–50 cm	Clay	5.8 °C; 544 mm	Soil coring	1.06–1.80^i^	Rytter [32]
		Fine root	2–3	0–70 cm	Sand (washed)	5.6 °C; 550 mm	Soil coring	0.22	Rytter [82]
		Root (1–2 mm)						0.43	
	P	Leaf	5	Surface	Loam	15 °C; 676 mm	Litter bag	4.28–5.27	Cotrufo [78]
		Leaf	6	Surface	Silt loam	25 °C; 60–70 % RH	Isotopes	1.25	Rubino [109]
		Fine root	2	0–15 cm	Sandy	9.5 °C; 726 mm	Soil coring	0.42	Berhongaray [81]

*MRT* mean residence time, *HCG* herbaceous crops/grasses, *SRWC* short-rotation woody crop, *M Miscanthus*, *SG* switchgrass, *W* willow, *P* poplar, *n/a* not available, *SWC* soil water content, *RH* relative humidity, *WFPS* water-filled pore space

^a^Temperature and hydrological conditions, where these are either mean annual precipitation (in mm) in the field or soil water content, relative humidity, water-filled pore space and matric potential (*in kPa*) in the laboratory

^b^Calculated from dry matter loss

^c^Incubated with earthworms

^d^Model parameter

^e^Model parameter, based on Gill and Jackson [104]

^f^One-millimetre mesh size

^g^Four-millimetre mesh size

^h^The range covers different varieties

^i^Calculated by mortality/growth ratio

**Table 3:** Change in soil organic carbon and retention in soil under herbaceous crops/grasses and short-rotation woody crops systems from different sources under different environmental conditions. The specific crop systems are *Miscanthus* (principally *M*. × *giganteus*), switchgrass and giant reed for HCG and willow and poplar for SRWC. Data not available or undisclosed is indicated (n/a)

System	Crop	Source	Age (year)	Depth (cm)	Soil texture	Conditions (MAT; MAP^a^)	Method	ΔSOC (Mg C ha^−1^ year^−1^)	Retention (%)	Reference
HCG	M	Leaf	3	Surface	Silt loam	15 °C; −80 kPa^c^	C analyser	0.40		Amougou [64]
		Rhizome	3	0–30	Silt loam	15 °C; −80 kPa^c^	C analyser	0.47		Amougou [64]
		Root	3	0–30	Silt loam	15 °C; −80 kPa^c^	C analyser	0.15		Amougou [64]
		All residue	3	0–30	Loamy sand	10.3 °C; 1,048 mm	Isotope ratio	0.60–0.72		Zimmermann [134]
		All residue	3.5	0–100	Clayey silt	11.0 °C; 1,042 mm	C balance	2.36	72	Anderson-Teixeira, [57]
		All residue	6	0–30	Sandy loam	13 °C; n/a	Isotope ratio	1.25–1.52		Zatta [86]
		All residue	9	0–100	Loamy sand	7.4 °C; 706 mm	MS	0.78	26	Hansen, [106]
		All residue	9	0–60	Silty clay loam	13.3 °C; 700 mm	CHNO; IRMS	2.08^d^		Cattaneo [140]
		All residue	14	0–30	Silty clay loam	10.3 °C; 704 mm	IRMS	0.41–0.46		Richter [80]
		All residue	14	0–60	Sand	9.3 °C; 830 mm	IRMS	3.20^e^	53	Dondini [45]
		All residue	15	0–30	Sandy loam	9.9 °C; 1,004 mm	MS	0.59	21.5	Clifton-Brown [19]
		All residue	16	0–100	Loamy sand	7.4 °C; 706 mm	MS	1.13	29	Hansen [106]
	SG	Litter	5	0–7.5	Silty clay loam	n/a; 723 mm;	CN analyser	0.89	41	Wienhold [77]
		BGB^b^	5	0–100	Sandy loam	n/a	n/a	1.10		Bransby [9]
		Root	3	0–90	Sand	10.5 °C; irrigated	IRMS	0.35	27 ^h^	Collins [47]
		All residue	2	0–30	Silt loam	16.0 °C; 1,180 mm	CN analyser	0.40–0.85		Garten [71]
		All residue	3.5	0–100	Clayey silt	11.0 °C; 1,042 mm	C balance	2.28	70.6	Anderson-Teixeira, [57]
		All residue	4	0–90	Silty clay loam	6.3 °C; 602 mm	CNS analyser	2.40^d^		Lee [75]
		All residue	7	0–40	Silty clay loam	9.9–11.4 °C; 856–1,092 mm	CN analyser	1.00–2.57	50.1^i^	Bonin [137]
		All residue	9	0–30	Silty clay loam	n/a	IRMS	0.66^d^		Follett [138]
		All residue		0–150	Silty clay loam	n/a	IRMS	2.05		Follett [138]
		All residue	23	0–60	Loam subsoil	10.5 °C; 914 mm	Combustion	3.71^f^		Al Kaisi [73]
	GR	All residue	9	0–60	Silty clay loam	13.3 °C; 700 mm	CHNO; IRMS	3.62^d^		Cattaneo [140]
SRWC	W	Litter	22	Surface	Clay	5.8 °C; 544 mm	Extrapolated^j^	0.17	23	Rytter [26]
		Fine root	22	0–50	Clay	5.8 °C; 544 mm		0.24	12	
		Fine root	2–3	0–70	Sand (washed)	5.6 °C; n/a	Modelled	0.22		Rytter [82]
		Litter and root	7	0–40	Silty clay loam	9.9–10.9 °C; 856–1,057 mm	CN analyser	1.14, 3.57^g^		Bonin [137]
		All residue	12	0–25	Loamy sand	9.3 °C; 595 mm	C analyser	0.22		Hellebrand [54]
		All residue	12	0–25	Loamy sand	9.3 °C; 595 mm	C analyser	0.34		
		All residue	5–19	0–45	Silt loam	9 °C; 981 mm	C analyser	−0.06		Pacaldo [143]
	P	Litter	22	Surface	Clay	5.8 °C; 544 mm	Extrapolated^j^	0.22	23	Rytter, [26]
		Fine root	22	0–50	Clay	5.8 °C; 544 mm		0.30	12	
		All residue	4–11	0–50	Clay loam	3 °C; 463 mm	C analyser	2.29		Arevalo [60]
		All residue	12	0–25	Loamy sand	9.3 °C; 595 mm	C analyser	0.23		Hellebrand [54]
		All residue	12	0–25	Loamy sand	9.3 °C; 595 mm	C analyser	0.53^d^		
		All residue	15	0–40	Sandy loam	10 °C; 417 mm	Wet oxidation	0.13^g^		Zhang [89]
		All	19	0–150	Clay	10.4 °C; 630 mm	Dry combustion	0.47^g^		Upson [92]

*ΔSOC* change in soil organic carbon, *HCG* herbaceous crops/grasses, *SRWC* short-rotation woody crops, *M Miscanthus*, *SG* switchgrass, *GR* giant reed, *W* willow, *P* poplar, *(n/a)* not available, *MAT* mean annual temperature, *MAP* mean annual precipitation

^a^Mean annual temperature and mean annual precipitation, unless stated otherwise

^b^Belowground biomass

^c^Matric potential (in kPa)

^d^Soil fertilised with N

^e^Including 0.81 Mg C ha^−1^ year^−1^ in soil micro-aggregates

^f^Re-cultivated subsoil

^g^Soils under short-rotation forestry

^h^Retention based on root input only

^i^Retention based on yield

^j^From Rytter [58]

For the synthesis of experimental evidence, the data were extracted from the articles’ tables and figures and scaled to a common unit (Mg C ha^−1^ year^−1^). We did not account for differences in temperature or other experimental variables. C inputs were either those measured directly or were estimated from accumulated dry biomass data, dividing by length of experimental interval and assuming a C content of 45 % unless authors stated otherwise (Table 1). The MRT of biomass C inputs was calculated from the measured fraction of mass lost during a defined incubation period, expressed in years assuming a linear response (zero-order kinetics). The non-linear decomposition (first-order) rates observed in laboratory incubations were scaled for temperature assuming a *Q*_10_ of 2, as discussed in the respective paragraph (Table 2). It is important to note that MRT usually represents residue decomposition rather than longevity. When the MRT was calculated from the ratio of new or dead plant parts to standing biomass by direct observation (i.e. roots), MRT represents longevity or mortality [31, 58]. We list retention factors of C inputs retained as SOC where given (Table 3).

## Experimental Evidence for Model Parameters

### Gross C Input Rates to Soil

Both HCG and SRWC can potentially deliver up to 40–50 % of the C they capture from the atmosphere to the soil as litter and BGB, which can vary from <1 to >4 Mg C ha^−1^ year^−1^ [59–61]. Several authors provide data on C inputs from PECs, generally discriminating between above- (litter) and belowground sources (roots and/or rhizome/stool). Such inputs differ considerably in the literature (Table 1), even for the same kind of residue, mainly due to environment but also due to differences in sampling and analytical methods. The reliability of some data is reduced when C inputs are approximated from biomass yield and an average C content of the residues [30, 62]. In general, fresh and easily decomposable matter, like fine roots and leaves, have a slightly smaller biomass C fraction (43 %) than woody material (46 %) [60, 62].

#### Aboveground Inputs: Leaves, Litter and Harvest Residues

Aboveground residues from HCG consist of fallen leaves and shoots (stems with leaves) which are seasonally produced before or left after harvest. They may accumulate on the soil surface forming a litter layer. The input range appeared to be quite large as it is assumed to be proportional to yield [63] but also depends on harvest dates [64]. C inputs have been variously measured as leaf litter accumulation on the surface [45, 57, 62, 65], as the difference between peak and harvested yield [19], and harvest residues [66–68]. On average, the C input to soil from HCG litter ranges from <1 to 3 Mg C ha^−1^ year^−1^. Detailed data on litter C inputs can be approximated from time series of dry matter measurements in an establishing [64, 65, 69] or mature crop [65].

Similar values exist for switchgrass [9, 17, 25, 43, 57, 67, 70, 71] which confirm the knowledge for *Miscanthus* and characterise differences for major land use alternatives. Litter inputs from leaves vary greatly under switchgrass due to management; they were highest in riparian buffer strips [72] and lowest under subsoil amelioration [73] and were affected by land quality [74–76] or water [47]. Litter additions were studied with regard to their effect on SOC fractions [77].

Under SRWC, litter production varies across species and genotypes, as leaf litter is proportional to LAI, and generally accumulates at the end of the season in a layer on the soil surface before being progressively incorporated into the top 10 to 15 cm of soil [78]. The range of C inputs from litter in SRWC (0.63 to 4.68 Mg C ha^−1^ year^−1^) is only slightly larger than those from HCG. Consistent across species and different locations, observed inputs seem to increase with stand age [60, 78], which is not reflected in long-term extrapolations [26]. There is also dieback of smaller stems especially during the first year [79] which can be a few percent of the biomass. Harvest method can have a profound impact on losses, but woody harvest residues <5 % seem achievable [22].

#### Belowground Inputs: Root and Rhizome

Data for C inputs from BGB are difficult to interpret, especially for roots because they are based on many single observations [45, 61, 62, 64, 80] rather than time series [26, 32, 81–83]. Due to differences in stand age, depth and sampling frequency inputs appear very variable [65, 66, 84]. On average, BGB inputs appear similar to that from litter under *Miscanthus*, switchgrass and willow but much smaller under poplar. There is some indication that inputs increase over time [60]. Inputs from roots may depend on soil texture and other environmental factors, but the dataset is too small to derive a rule; however, longevity of roots and their turnover depends on root diameter [30, 31].

For HCG, authors differentiate the C contribution to soil between those from the rhizome, a storage organ often present in grasses used as biomass crops, and from fine, fibrous roots. The latter contributes less than 25 % of the apparent annual BGB input under *Miscanthus* (Table 1 and Fig. 4). BGB is concentrated in the upper part of the profile [61]; in spite of large amounts of roots under *M. giganteus* observed down to 1 to 1.8 m [57, 61, 66, 80, 84], more than 50 % were in the top 30 cm. The C inputs from *Miscanthus* rhizomes (1.01 to 2.66 Mg C ha^−1^ year^−1^) were generally greater [57, 61, 66, 80, 85] than those from roots (0.19–0.86 Mg C ha^−1^ year^−1^, Table 1). For switchgrass, more C was allocated to the roots than to the rhizomes [25, 66, 71]. Some studies did not separate rhizomes from roots [9, 45, 57, 86] or ignore them completely [47, 61, 67, 72].

**Fig. 3:**
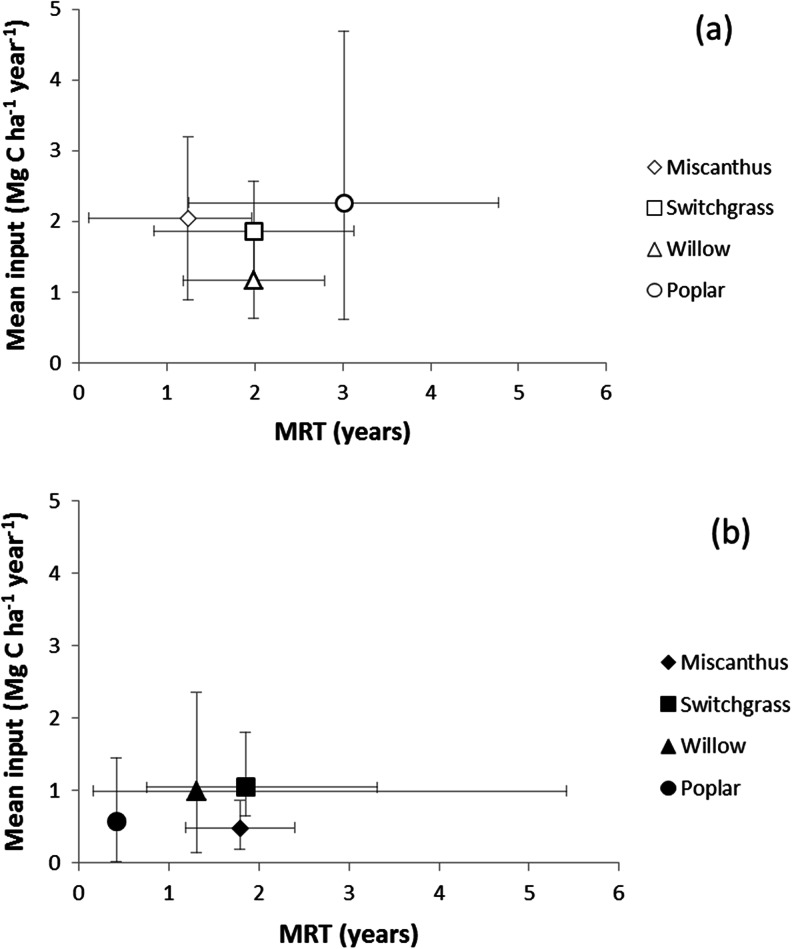
Mean C input and mean residence time (*MRT*) from **a** leaf litter and **b** roots of perennial energy crops as derived from the data in Tables 1 and 2, respectively. The *bars* show the maximum and minimum values in the range

Fewer data were available for SRWC systems due to the practical difficulties of excavating and studying tree roots. For different species in SRWC systems, 25–35 % of total tree biomass was found in the roots [87], with 10 % specified as fine roots, confirmed for a 10-year-old poplar plantation [5]. Several authors investigated fine roots from willow and poplar grown as SRC [5, 30, 81, 82, 88, 89] and SRF [72, 90–93], values for the latter being lower (Table 1). Quantification of fine roots is challenging, and indirect methods were proposed although roots concentrate in the upper 5–10 cm of soil [94]. Variable fractions of willow biomass can be found belowground dependent on stand age and soil texture [58]; 48–58 % of total biomass was allocated in the first year and 40 % allocated in succeeding years [26]. Observed values of C input from fine roots of SRWC poplar range between 0.46 and 1.28 Mg C ha^−1^ year^−1^, and between 0.16 and 1.45 Mg C ha^−1^ year^−1^ can be found in coarse roots (Table 1). It was suggested that about 20 % of SRC poplar biomass was allocated to structural roots [95] which may contribute significantly to biomass C, whilst fine roots contribute to SOC due to their faster turnover compared to structural components [26, 82]. Average fine root inputs are higher under willow than poplar (Fig. 3b).

#### Root Exudates

Little is known about the effect of root exudates on SOC. Only two papers have been published with root exudate data from *Miscanthus* [96, 97]. Kaňova et al. [97] have quantified and qualified exudates from roots of 15-year-old plants. Based on their hourly flux measurements, C exudate inputs from *Miscanthus* could be up to 0.5 g g^−1^ living root produced annually. Based on root biomass data (Table 1), C exudates and 2-year MRT (Table 2) and assuming 210-day season with an average 12-h day length roots could contribute between 0.4 and 1.7 Mg C ha^−1^ year^−1^ to the C balance. Hromadko’s data [96] were an order of magnitude larger than those presented by Kaňova [97] which seems unrealistically large. Further investigations showed that these root exudates contributed to soil respiration and may affect SOC pools in multiple ways.

C measured in the rhizosphere of 2-year-old willows, grown in the glasshouse using stable isotopes (^14^C) pointed to high rhizodeposition of C in older plants which was not proportional to the root production [98]. This C turned over very fast and only 2 % was found in the soil microbial biomass. However, 11 % of rhizodeposits were retained in the soil organic matter. For a 3-year-old poplar stand, it was suggested that C from fine roots (including exudates) could stimulate the microbial biomass and induce turnover of already stored SOC [99]. Rhizodeposition and root exudates can cause a priming effect on the turnover of C stored in the soil [100], which could increase soil C emission [101, 102] and lower the sequestration rate. However, living roots can decrease (50 %) or increase (380 %) SOC mineralisation rates causing losses equivalent to the root C input to soil, the range depending on plant species and age [103]. The loss of existing SOC increased with root biomass in a poplar plantation subjected to free air C enrichment (FACE) [99] but did not appear to impair SOC increase.

In summary, gross inputs from leaf and stem litter and harvest residues are more tangible than from BGB. The production of fine roots and root exudates could vary greatly between species, genotypes, management and the age of the perennial and is tedious to assess. There is little quantitative knowledge about the site-specific interaction between the size of the residue inputs and SOC and how they were affected by management (e.g. fertiliser, soil fertility, harvest). Inputs from perennials are continuous, vary over their life time and their variable size and composition are likely to affect their decomposition.

### Residue Dynamics and Turnover Rates

#### Longevity, Mortality and Decomposition

The impact of C inputs from PEC systems on SOC cannot be assessed without quantification of the longevity or mortality before turnover of each component (leaves, shoots, rhizome, stool and coarse and fine roots). The assessment of decomposition rates of BGB of herbaceous and woody species presents a conceptual problem. BGB can die and start to decompose at different ages with different decay rates dependent on size and environmental conditions [31, 104].

*Miscanthus* rhizomes and roots are potentially the most easily decomposable parts [64], but in its [19] function as a storage organ, the carbohydrate and crude protein content of the rhizome changes seasonally to support regrowth of the plants in spring [105]. With age and distance to the ‘growing edge’, old parts of the rhizome lose their vitality, become inert and start decomposing whilst the younger parts remain alive and store C for quite a long time before this can be available to soil. From changes in SOC, it was hypothesised that C from dead rhizomes and roots becomes available only 7 years after *Miscanthus* planting [1]. From evidence on *Miscanthus*-derived SOC in the surface horizon (13 and 31 % in a 9- and 16-year-old stand, respectively), a rhizome longevity of 8 years was postulated [106]. From the composition of BGB under a 15-year-old *Miscanthus* crop (19 % dead rhizome, 66 % live rhizome and 15 % live root [19], one could approximate a relative annual death rate of 3 %. Longevity of roots was found to vary greatly [30], likely to be controlled by diameter [31].

The longevity of aboveground plant parts is clearly defined by their abscission, but the decay of aboveground litter depends on its incorporation into the soil [107] and accessibility [34]. The decomposition rates established by using litter bags will underestimate the flux of C from the residues to the soil pools, as shown for *Miscanthus* [108] and poplar [78, 109]. Earthworms clearly enhance the incorporation of residues into the soil and accelerate their decay [110, 111].

#### Factors Affecting Decomposition and Mean Residence Time

In general terms, litter decay is proportional to its nutrient content and inversely proportional to lignin content [64]; it is also controlled by nutrient availability (particularly N) in the soil [112, 113]. The C/N ratio of *Miscanthus* litter increases from 28 to 34 in leaves at the end of season to 43–52 [65] or even >100 [110] in mature litter. This is likely to reduce turnover rates of mature *Miscanthus* litter [108, 114]. Lignin, however, seems to physically control decomposition of the litter by reducing the surface available for enzymatic attack [4]. Litter rich in lignin and poor in N was more sensitive to temperature changes than protein-rich litter [38] as shown for *Miscanthus* residues [108, 114]. In vitro decomposition of *Miscanthus* litter was affected by N added as pig slurry [115] rather than N added as fertiliser [116]. This suggested the importance of microbial biomass colonising litter [117, 118].

Litter mineralisation depends also on the soil fauna [119, 120], which may vary according to location [113]. It is difficult to estimate in situ litter decomposition, both in SRWC [78, 109] and in HCG [108]. The commonly used litter bag method [64, 69, 78] prevents physical incorporation of the debris into the soil and does not allow access of larger biota (e.g. earthworms). C losses may be solely a function of microbial respiration within the litter bag [109], and other fundamental soil processes affecting turnover, such as adsorption to clay and occlusion within the soil matrix [35, 121], were not accounted for. The use of isotopic methods gives a much better account for the in situ complexity of soil systems [109].

#### Residue Turnover and Mean Residence Time

Available literature on decomposition rates for PECs was patchy, and usable data were limited to 15 experimental papers (Table 2). Discrepancies regarding the experimental evidence for the same residue type were found due to the methods used and the experimental setup. Most obvious is the effect of temperature on the MRT of leaf *Miscanthus* litter derived in litter bags and laboratory incubations [62, 64, 69, 108, 122].

Potential decomposition rates of *Miscanthus* residues, both in situ (litter bags) and by laboratory incubation, decreased in order from young rhizome to litter and roots, with MRT ranging from 1.2 to 2.4 years [64, 69], similar to other results [62]. About 35 % of leaf C was stabilised as recalcitrant plant debris or as microbial biomass [64], but the amount of C stabilised in humus remains unknown. MRT for leaves, alone or combined with other aboveground residues, ranged between 0.9 and 3.19 years and were longer under drier [62] and colder conditions [108]. Turnover rates can be scaled to standard moisture and temperature conditions to assess the effect of residue age. A microcosm experiment showed that free access by soil fauna increased litter turnover by an order of magnitude [110] compared to those observed in litter bags [64, 123].

Litter bags were also used to estimate long MRT for switchgrass residues [4] which is complemented by literature on SOC turnover affected by root size [124] and change of MRT of SOC fractions [25, 47]. Model estimates [8] were based on a global study [104]. In situ decomposition rates of roots could be low as both switchgrass [70] and *Miscanthus* [61] roots penetrate into deeper, cooler and less aerated soil layers. Coarse and dense roots of old undisturbed grassland turn over much more slowly (0.92–1.32 years) than fresh inputs [125].

In SRWC systems, similar discrepancies due to experimental procedures occur: Only 15–18 % of the original mass was lost from litter bags over 8 months [78], whilst a loss of 80 % of the same litter was estimated during an 11-month period using ^13^C-labelled material [109]. These losses translated into MRTs about four times longer (4.3–5.3 years) than those determined from isotope ratios (1.15 years) and about twice those calculated when accounting for the 10 °C difference in incubation temperatures. In situ decay rates of leaves were much higher when using large instead of small mesh litter bags [111]. Leaf material accessible to earthworms almost completely disappeared from the large mesh litter bags within 1 year (95 %; MRT ∼1 year), whilst material accessible only to fungal decomposition only lost between 40 and 80 % (MRT 2.5 to 1.25 years).

It is disputed whether fine roots contribute most to SOC in woody systems due to their relatively rapid decomposition [5, 99, 126]. However, fine willow [58] and poplar roots [81] were characterised by a high turnover rate (Table 2). However, the average production of fine roots under poplar was smaller compared to willow (Fig. 3b; 0.56 vs. 0.99 Mg C ha^−1^ year^−1^). Turnover times for willow roots [127] determined from a mass loss (15–25 %) in litter bags were much longer than those determined using other methods (Table 2).

The longevity of fine willow roots can range between 55 and 350 days [32, 58, 83]. Similar values were found for fine poplar roots [30]. Coarse roots (CR) represent a variable fraction of the root biomass (10 and 40 %; [58, 99]), but much smaller values emerged recently [82]. Very coarse, anchoring roots are likely to have a long MRT (e.g. lifetime of the stand), but there are no reports on decomposition of those [81, 82, 99].

In summary, a good number of contrasting results regarding residue turnover was compiled from the literature, which enables initial parameterisation of plant–soil interaction and C flux models (see below). However, there is relatively little known about the in situ turnover of live rhizomes and coarse roots as longevity becomes an additional level of complexity. The large range of MRT reflects the experimental conditions, long MRT being an artefact of litter bag usage. However, a small number of studies using isotopes [109], model systems [110, 111] and field sampling [32, 81, 82] allow their scaling to in situ rates.

### Carbon Retention and Storage in Soil

The factors affecting the soil C balance are numerous and vary depending on climate, soil conditions and crop management, e.g. harvest date and residue removal [62, 128]. Integrated studies on C enrichment, e.g. POPFACE, have shown that increased atmospheric CO_2_ can induce a decrease of SOC [78] attributed to a priming effect [99, 102, 129, 130]. However, physical protection and chemical adsorption, both controlled by soil texture (e.g. clay content and cation exchange capacity), may play an important role for long-term C retention [4, 34, 35, 101]. C retention is the net result of annual gross inputs/production, mortality and turnover of the components.

Figure 3 synthesises the average gross inputs (Table 1) and their MRT (Table 2) for each residue type, distinguishing litter and roots. It is important to realise that inputs were often based on snapshots, whilst detailed time series were the exception, especially with regard to root production and turnover [32, 81, 82]. Grasses and poplar have similar C inputs from AGB litter, larger than willow SRC (∼2 vs. 1 Mg C ha^−1^ year^−1^) with a wide range of MRT (<1 to >4 years), some of which may be overestimated. Within the SRC data, there is more quantitative evidence from poplar [60, 72, 78, 79, 99, 109] than for willow [58, 111, 131].

**Fig. 4:**
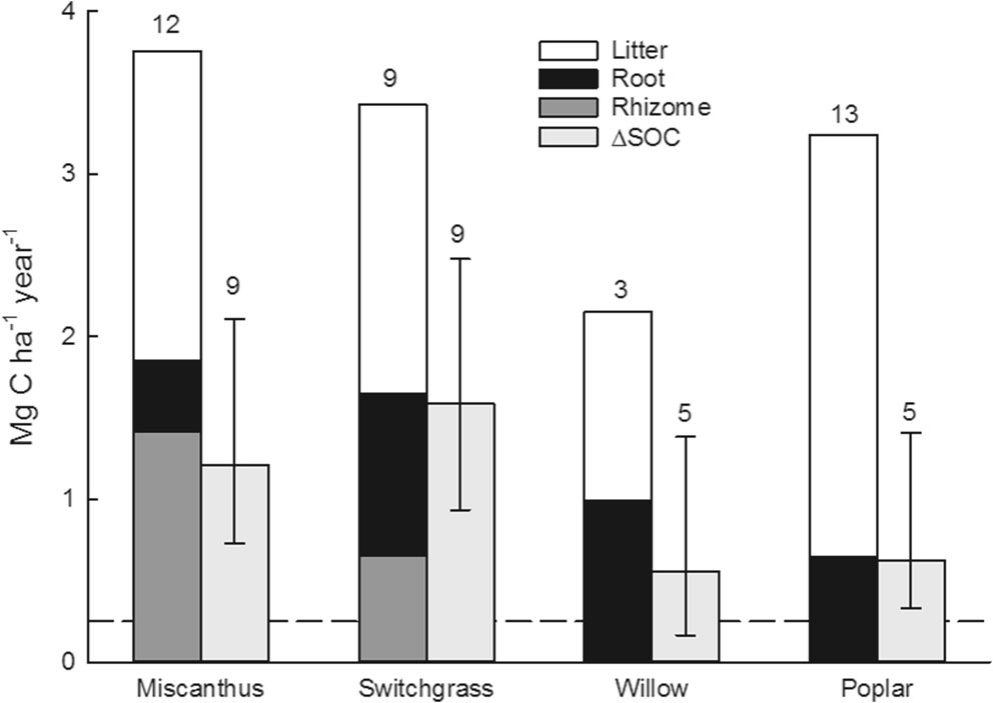
The mean input of carbon (*C*) (*stacked column*) and the mean change in soil organic C (Δ*SOC*) (*single column*) from perennial energy crops as derived from the data in Tables 1 and 3, respectively. The *bars on the ΔSOC* column show the maximum and minimum values in the range, and the *numbers above all the columns* give the numbers of references considered

For the BGB, inputs from HCG roots and rhizomes needed to be separated, which was not always done [45, 57, 86, 106]. Annual C inputs from *Miscanthus* roots are about half that of switchgrass with similar turnover time. Rhizome inputs from switchgrass were much smaller than from *Miscanthus* which explains the evidence that switchgrass sequestered twice the amount of SOC (Fig. 4). In spite of methodological uncertainty regarding turnover of fine roots, the MRT of HCG was about four to five times longer than that of SRWC (Fig. 3b). The annual average C input to the soil as fine root under SRWC is between 0.4 and 1 Mg C ha^−1^ year^−1^, but their fast turnover is diminishing the contribution to SOC storage and retention to about 10 % [82]. However, what really makes the difference between the two systems in addition to the difference of SRC fine roots turnover is the dynamic of the rhizome.

Although annual rhizome inputs are similar to those from litter, its MRT is large due to its longevity [1, 68] which delays their decomposition and incorporation into SOC. The analogue in the SRWC system is to be found in the coarse roots (1–2 mm) with longer MRT [82] or woody roots, the stump or stool [4] for which no evidence of turnover and retention exists.

#### Evidence for Long-Term C Storage Change

Potential, default C sequestration rates for arable crops, grassland and forests have been estimated to be 0.3, 0.5–0.7 and 0.1–0.2 Mg C ha^−1^ year^−1^, respectively [132]; others found 0.33 Mg C ha^−1^ year^−1^ for both grassland and forest systems [133]. The net SOC storage change under PECs observed in most recent studies exceeds these values (Table 3). The C effectively sequestered under *Miscanthus* (% retention) ranges between 21 % [19] and ∼70 % [57] of the gross C inputs. Across different environments and time periods, quite similar net storage changes could be found (3 to 15 years) [19, 134]. However, a comparison across similar environments indicates that with increasing age (9- to 16-year-old *Miscanthus* stands), the amount of annual SOC increment rises by more than 40 % [106]. High rates of SOC were sequestered in a soil very low in C [135] compared to those established on grassland sites (e.g. [19, 86]. Assuming steady-state C inputs [135] could lead to an underestimation of MRT [136]. Yields and litter inputs increased during the establishment phase [65] and the postulated delay for rhizome decomposition [106] contradict such assumptions.

Under switchgrass, a large number of studies [8, 9, 47, 57, 75, 77, 137, 138] showed sequestration rates, which were similar (Table 3) but higher on average (Fig. 4). Retention rates were also higher on average (50 %), probably due to large fraction of root inputs.

In-depth analysis supports the concept of an initial resilience phase of SOC after LUC to PEC in which *Miscanthus*-derived SOC did not fully replace the continuing turnover of resident C during early establishment. Especially when planted into grassland soils [86, 139], the initial residue input and turnover rates from PEC are too low to compensate for the loss. Zimmermann et al. [141] also showed that BGB has a greater effect on SOC than litter inputs, possibly due to delayed incorporation of the latter. Early estimates of long-term SOC stock changes based on a single observation [19, 135] need to be revised using a time series with early phase evidence [86, 134, 139] and accounting for spatial heterogeneity [46, 57, 80, 86]. Sequestration rates are not predictive when based on single early or late observations [19, 135, 140], average C inputs and turnover rates.

C inputs originate from and feed into pools of different stability [45, 141], and initially, C is stored as particulate organic matter (POM) [139], which is a relatively slow SOC pool under switchgrass [25, 47]. This could explain the lack of real enrichment of recalcitrant or mineral-associated SOC during the first 4 or even 6 years under *Miscanthus*, especially when planted into former grassland [86]. These results are in line with findings for switchgrass [25]. Losses of SOC under *Miscanthus* genotypes with higher BGB were suggested to indicate higher soil respiration rate and C turnover induced by root exudates, causing a priming effect [86]. Considering the enrichment in the labile fraction [86, 135, 139], it was suggested that at least 20 years was needed to evaluate the impact of *Miscanthus* in terms of sustainable SOC enrichment, which contradicted earlier estimates [19, 45, 106, 135]. With regard to verifying the simulation of sequestration dynamics, it seems necessary to have observations for inputs and changes of both total and specific SOC fractions along the whole growth period. For switchgrass, similar average inputs appear to cause an overall 25 % higher average stock change (∆SOC, Fig. 4) than *Miscanthus* (1.59 vs 1.21 Mg C ha^−1^ year^−1^), likely due to the lower root inputs from *Miscanthus* (Fig. 3b).

Based on long-term field trials for SRWC and accounting for all residues, overall lower C sequestration rates were calculated for willow and poplar (Table 3; average 0.56 and 0.63 Mg C ha^−1^ year^−1^, respectively) than for HCG systems. An increased annual SOC storage change was observed in response to fertilisation, particularly in poplar stands [54]. These low-storage changes were supported by findings for SRF using labelled C [142], which also showed that new C accumulated in SOC pools was characterised by high turnover rates. However, ∆SOC under SRF/SRC changed within 11 years from an initial loss of 0.74 during the first 2 years to an increase of 5.82 Mg ha^−1^ year^−1^, during the last 2 years [60]. For Table 3, we interpolated after the initial loss an average annual SOC gain of 2.29 Mg ha^−1^ year^−1^ between years 4 and 11, which over the whole period would have been an increase of only 1.87 Mg ha^−1^ year^−1^. These numbers are similar to the average gains reported for SRC poplar earlier (1.63 [68] to 2.43 C Mg ha^−1^ year^−1^ [10]). These sequential long-term measurements are essential to extrapolate results from short-term controlled experiments to long-term field rotations (e.g. Rytter [26, 58]; Table 3). New data show high sequestration rates for switchgrass on arable soils [137] but no long-term change for SRC-willow on former grassland soils [143].

In conclusion, the variability of ∆SOC under PECs reflects the uncertainty due to the limited periods of measurements, a series of simplifying assumptions (linearity, steady state) and environmental variability (see above). Based on Table 3, the C retention is higher in herbaceous (*Miscanthus*) than woody PEC systems (21–72 vs. 12–23 % retained of original input), which is confirmed by comparing independent averages (Fig. 4). Discrimination between inputs from above- and belowground residues (Tables 1 and 2) suggests input- and plant-specific C retention factors (Fig. 3). The view that roots contribute more to SOC than aboveground sources [100] seems to be reflected in Figs. 3b and 4, and recent data showed a tight relationship between root and *Miscanthus*-derived SOC [80]. The average sequestration rates (Fig. 4) are largely based on all residues and require further analysis in terms of component-specific contributions.

#### Decomposition and Stabilisation of SOC Pools

The stabilisation of SOC pools depends essentially on the relation between soil temperature [41], chemical composition [144] and physical protection. The latter defines their kinetic properties and temperature sensitivity [145, 146]. The least decomposable residues seem to be the most sensitive to temperature [120]. Particulate (POM) and mineral-associated organic matter (MOM) are formed in the soil, which stabilise SOC, e.g. limiting the access of soil microbes [25]. Hansen [106] found 65 % of *Miscanthus*-derived C stabilised in POM. Occluded POM is particularly stable, and root C has been found to accumulate in this pool even under arable crops [147]. The amount of C occluded, which is considered as a main index of long-term sequestered C in soils under forest [148] as well under *Miscanthus* [11], remains to be quantified. The intra-aggregate fraction of POM [45, 139] is small but could be a close approximation.

Lignin as a biomass component together with polyphenols reduces decomposition rates [149]. However, lignin decomposition rates are highly variable depending on soils and land use and are not necessarily linked to the stability of the derived soil C pool [146, 149]. Recalcitrant SOC components, like lignin, increase with depth and clay content due to chemical binding [35, 149]. The bio-physicochemical complexity of SOC could explain its turnover variability under PECs. *Miscanthus*, whose litter is higher in lignin than other grasses [69], seems to reduce the turnover rate of existing SOC by increasing the insoluble C fraction resistant to microbial attack [116, 150]. On the other hand, a priming effect was suggested for *Miscanthus* root exudates [86], and a two- to fourfold increase in the mineralisation rate was reported after establishment of switchgrass [43]. For forest systems [142], it has been shown that new C inputs from plant residues can enter relatively fast decomposable SOC pools, particularly if the soil had a low clay content [148]. Under SRWC systems, the predominance of decomposable fine roots could explain the initial loss of SOC [60] and higher losses under FACE [99].

#### CO_2_ Emission and Other Losses

In general terms, C losses from soil come from root respiration (ca. 20–40 % of plant-fixed C; [151]), microbial respiration (40 % of SOC in decomposable pools [151]) and leaching of DOC. Pieces of evidence for losses from SOC pools using measurements of CO_2_ emissions are considered essential to explain the complex dynamics of C fluxes [66] and were included in several studies [48, 49, 52, 53, 55, 57]. Earlier estimates of C emissions from PECs are usually derived from models, and typically very little CO_2_ is emitted from *Miscanthus* stands of different ages [59]. N_2_O emission was 4 to 6 Mg CO_2_-equivalent higher after grassland conversion to willow and poplar, but CO_2_ fluxes were 30 to 40 % less than on the grass reference [55]. Conversion from arable to *Miscanthus* or switchgrass confirms higher CO_2_ emissions under grassland (e.g. prairie) [57] reflecting its higher biological activity which eventually increased SOC stocks. N fertilisation in switchgrass actually reduced the GHG emission per unit harvested biomass [53]. Under SRWC systems, coppicing itself can result in a 50 % increase in CO_2_ emission, mainly due to live root respiration [152]. Root respiration has also been found to increase in SRWC systems under enhanced atmospheric CO_2_ concentrations [130], which seems to be generally valid for FACE experiments under older (12–16 %) and recently established (22–46 %) systems [153].

DOC typically comprises compounds of low molecular weight. Exudates (the main source of DOC) can be adsorbed by the soil, abiotically decomposed, volatised or leached. DOC could, in spite of its small size, be of relevance under PECs due to the extensive root system and large rhizome biomass. To date, there have been no specific studies published to quantify DOC in PEC systems. De Neergaard et al. [98] found that willows allocate between 1 and 10 % of plant C to root turnover and exudation, similar to those found for other plants [154]. DOC persistence or loss by leaching was mentioned for *Miscanthus* only qualitatively [155]. However, under grassland, 20–30 % of DOC was rapidly decomposed by microbial respiration, <5 % was retained in the soil, whilst the rest was assimilated by the soil microbial biomass [156]. Similar conclusions were reached for DOC in forest soils [157], where the soil microbial biomass assimilated 70 % of the DOC and respired the remaining 30 %.

In summary, under PECs, the main difficulty in assessing C turnover and retention is the uncertainty regarding the physical and chemical stability of SOC pools. It is the stability of these pools which defines the sustainability of soil C sequestration and eventually the effective C neutrality of the system [125, 142]. The half-life of >25 years considered for a C pool to be ‘inert’ [121], and similar times as a criterion for SOC sequestration [5, 35, 151, 158] seems rather short. They are in contrast to much longer times assumed for the ‘inert’ and stabilised humus fractions in SOC turnover models [159, 160]. From these considerations, the postulate arises that sequestration can only be assessed if a time series of long-term measurements of these pools are available (e.g. maintaining monitoring and demonstration trials for PECs).

## Modelling Approaches Adopted for SOC Under PEC Systems

When assessing the effects of land use change on SOC turnover, model complexity is dependent on data availability and process understanding at different scales. For PEC systems, the evidence base is still emerging and is too sparse to model the process dynamics, let alone to predict a state of equilibrium [8]. For large-scale applications, process-based models were simplified, based on a series of assumptions (e.g. steady state) and controlled by empirical inputs (e.g. yield maps [63]). In the following, we will review process-based models of varying degree of complexity/empiricism and their actual application to PEC systems. We will use an expanded semi-empirical process model to rank the importance of the key inputs and parameters presented above.

### Process-Based Models

There are several papers which review models applied to the C balance [14, 15] or GHG emissions [12, 16, 30] under PECs [161]. The most recent review on models relevant to PEC systems screens complex agro-ecosystem models with a focus on N_2_O emission [15]. CENTURY [160] and RothC [159] were identified as especially important. The principles of C fluxes under land use change from arable to grassland and forestry systems certainly apply to the transition to PEC as shown recently using a hybrid C and N model including routines of CENTURY [162].

A total of 25 papers modelling C in PECs systems were published between 2000 and 2013: 16 used RothC and derived models, five used CENTURY or DayCENT and two used both models (Table 4). Two papers dealt with a generic description of the C balance under poplar [10] and switchgrass [8]. The popularity of RothC may lie in the minimal data requirement, its adaptability, which allows its integration, and the easier calibration of residue pools compared to CENTURY [163]. Authors have modified and coupled RothC by adding and calibrating new specific soil and residue C pools, accounting for tree [164] and crop [165] residues. RothC has been coupled with specific crop growth and litter degradation models to simulate SOC dynamics under *Miscanthus*, willow and poplar SRC [166]. Dondini et al. [45] applied a soil fractionation technique [141] to improve the calibration of SOC pools and to account for the recalcitrant nature of plant residues. Within FullCAM [166, 167], RothC was used in woodland systems by applying debris partitioning and decomposition rates measured in agricultural soils. However, previous work [168] showed that the C cycle in forest systems needed a chemical model for litter decomposition to integrate RothC with plant residues. They also suggested locally calibrated decomposition rates to account for soil temperature and water content gradients [166]. However, the question is whether a litter layer is mandatory in a SRWC system when managed as SRC, as surface litter in spite of its chemical composition seems to be incorporated within a season [111].

**Table 4:** Use of RothC and CENTURY models and derived routines in simulating C dynamic under herbaceous crops/grasses (HCG) and short-rotation woody crops (SRWC) grown as PECs and similar systems

Scale	System	Species	Soil	Location	Model	Objective	Duration	Output kind	Output unit	Paper
Laboratory	HCG	*Miscanthus* ryegrass	Sandy	DK	CENTURY	Rate calibration	n/a	ΔSOC	% C loss	Foereid [116]
		Mix grass	Quartz sand	n/a	RothC	Rate calibration	n/a	Decomposition rate	Year^−1^	Hoffmann [113]
		Alpine grass	Leptosol	CH	RothC	Rate calibration	Equilibrium	ΔSOC	Mg C ha^−1^	Leifeld [174, 175]
		Alpine grass	48 soils	CH	RothC	Pool calibration	n/a	ΔSOC	Mg C ha^−1^ year^−1^	Zimmermann [141]
Field	HCG	*Miscanthus*	Sandy loam	IRL	RothC	C sequestration	14	ΔSOC	Mg C ha^−1^ year^−1^	Dondini [135]
		*Miscanthus*	n/a	USA	DAYCENT	Mod. comparison	9	ΔSOC	C g m^−2^	Davis [59]
		*Miscanthus*	Clay loam	PL	CENTURY	C sequestration	2	ΔSOC	Mg C ha^−1^ year^−1^	Poeplau [11]
		*Miscanthus*	Different soils	NL; DK; CH; D	RothC	C sequestration	>10	ΔSOC	Mg C ha^−1^ year^−1^	Poeplau
		*Miscanthus*	Sandy loam	UK	RothC	C sequestration	7	ΔSOC	Mg C ha^−1^ year^−1^	Zatta [86]
		Alpine grass	Silty loam	CH	RothC	C sequestration	6	ΔSOC	C g m^−2^	Niklaus [129]
		Grass	Different soils	IRL	RothC	C sequestration	40	ΔSOC	Mg C ha^−1^ year^−1^	Xianli [163]
		Switchgrass	n/a	USA	DAYCENT	Mod. comparison	9	ΔSOC	C g m^−2^	Davis [59]
	SRWC	Olive	Clay loam	E	RothC	Management	30	ΔSOC	Mg C ha^−1^ year^−1^	Nieto [164]
		*Pinus*	Chromosol	AUS	RothC	Management	20	ΔSOC	Mg C ha^−1^	Paul [167]
		*Pinus*	Sand to clay loam	AU	RothC	System analysis	1–2	Litter loss	% loss	Paul [166]
		*Pinus*		AU	RothC	System analysis	40	ΔSOC	Mg C ha^−1^	Paul [168, 172]
		*Pinus*	Sandy soils	E	Both Mod	Mod. comparison	35	ΔSOC	C g m^−2^ years^−1^	Romanya [188]
		Willow	Clay loam	PL	CENTURY	C sequestration	2	ΔSOC	Mg C ha^−1^ year^−1^	Borzecka [186]
		Willow	Clay sandy loam	UK	CENTURY	C sequestration	3 and 24	ΔSOC	Mg C ha^−1^ year^−1^	Grogan [189]
Regional GIS	HCG	*Miscanthus*	Soil map	England and Wales	RothC	C sequestration	3	TOC	Mg C ha^−1^ year^−1^	Hillier [63]
	SRWC	Willow, poplar	Soil map		RothC	C sequestration	3	TOC	Mg C ha^−1^ year^−1^	[63]
		Willow, Poplar	Soil map	Scotland	DAYCENT	GHG emission	6–30	ΔSOC	Mg C ha^−1^ year^−1^	Shibu [162]
		Forest	Soil map	H	Both Mod	Mod. comparison	Eq	ΔSOC	Mg C ha^−1^	Fallon [119]

*DK* Denmark; *CH* Switzerland; *IRL* Ireland; *USA* United States of America; *PL* Poland; *NL* The Netherlands; *D* Germany; *UK* United Kingdom; *E* Spain; *AUS* Australia; *H* Hungary

#### C Dynamics as a Function of Soil Depth

The release of C from litter decomposition is a function of different factors, mainly controlled by its incorporation into the soil and by soil depth, which induces temperature and soil moisture gradients. CENTURY and APSIM decrease the decomposition rate of litter accumulating on the soil surface. Soil water dynamics are considered a key parameter in all SOC models applied to PECs [14, 15]. The models APSIM, CENTURY and STICS include a component for litter decomposition using a rate-modifying function for soil depth, which account for changes of temperature and moisture along the soil profile. Jenkinson and Coleman [40] introduced a similar empirical coefficient to reduce SOM decomposition in the subsoil. In addition, decomposition of old SOM can be affected by root priming [103], which varies with root density along the soil profile and crop C balance [99]. In summary, depth-dependent decomposition has been implemented by introducing (i) water and heat flux models [14, 169] or (ii) empirical coefficients [40] or (iii) including C transport, i.e. diffusion and adsorption of DOC [42].

#### C Pools and Their Decomposition Rate

Most process-based SOC models assume that degradation of residues from biomass can be represented by a continuum or two or more pools of plant or soil OM of similar chemical composition and degradability [161]. Each pool decomposes following first-order kinetics integrated with temperature and moisture functions. The use of more pools implies more calibrating parameters which improve long-term prediction [8] but increase the uncertainty of the model. Multi-pool or compartment models present challenges when applied to ligno-cellulosic (LC) plants. First, chemical or physical identification of specific pools with defined decomposition rates is very difficult [141, 163]. Second, it has never been proven experimentally that a multi-pool chain model can represent the decreasing decomposition of residues due to chemical and physical protection [118, 148, 170]. Finally, non-linear interactions between decomposition rate and temperature can exist for different pools, with the effect being greatest at lower temperatures and for ‘slowly decomposable’ compounds but least for ‘old’ SOC [14]. Further, C turnover derived from laboratory incubation of soil and residues overestimate those in undisturbed, natural systems [168, 171, 172]). Simulations were greatly improved when decomposition rates were calibrated using labelled C measurements [173–175] and applied to the experimental evidence presented for poplar litter [78, 109]. Decomposition rates of LC residues can vary with temperature, or through interaction between temperature and ‘recalcitrance’, which could depend on their chemical composition [4, 109]. Recalcitrance can also be modelled using a delay time of decomposition in dependence of accessible soil pore space, which simulate the physical protective effect of soil from microbial activity [176]. Other research differentiated inter- and intra-aggregate and particulate OM to derive pools fitting the different model concepts [45]. In perennials, however, biological protection (longevity) of coarse roots, stool and rhizomes (see above) is a major conceptual challenge that has not been met.

### Modelling Using Disaggregated C Inputs

So far, modelling SOC under biofuel crops has mainly relied on C inputs approximated from crop yields [19, 45, 86, 139]. A generalised link between yields and residues is plausible [63]. However, the assumption of another 10 % of roots to be accounted for as residue input [135] is not supported by any evidence. It is clear from the experimental evidence synthesised above (Tables 1 and 3 and Fig. 4) that the assumption of a single input source of residues may not be adequate to describe the C turnover and retention in the PEC soil system. Considering a single input source does not account for the surface accumulation of the litter and the longevity of the BGB. Therefore, we expanded RothC [159] in order to receive disaggregated C input fractions (litter, rhizomes, roots and root exudates) to test the importance of this new assumption and to explore the effect of different residue turnover rates (Table 2) on the change of SOC.

**Fig. 5:**
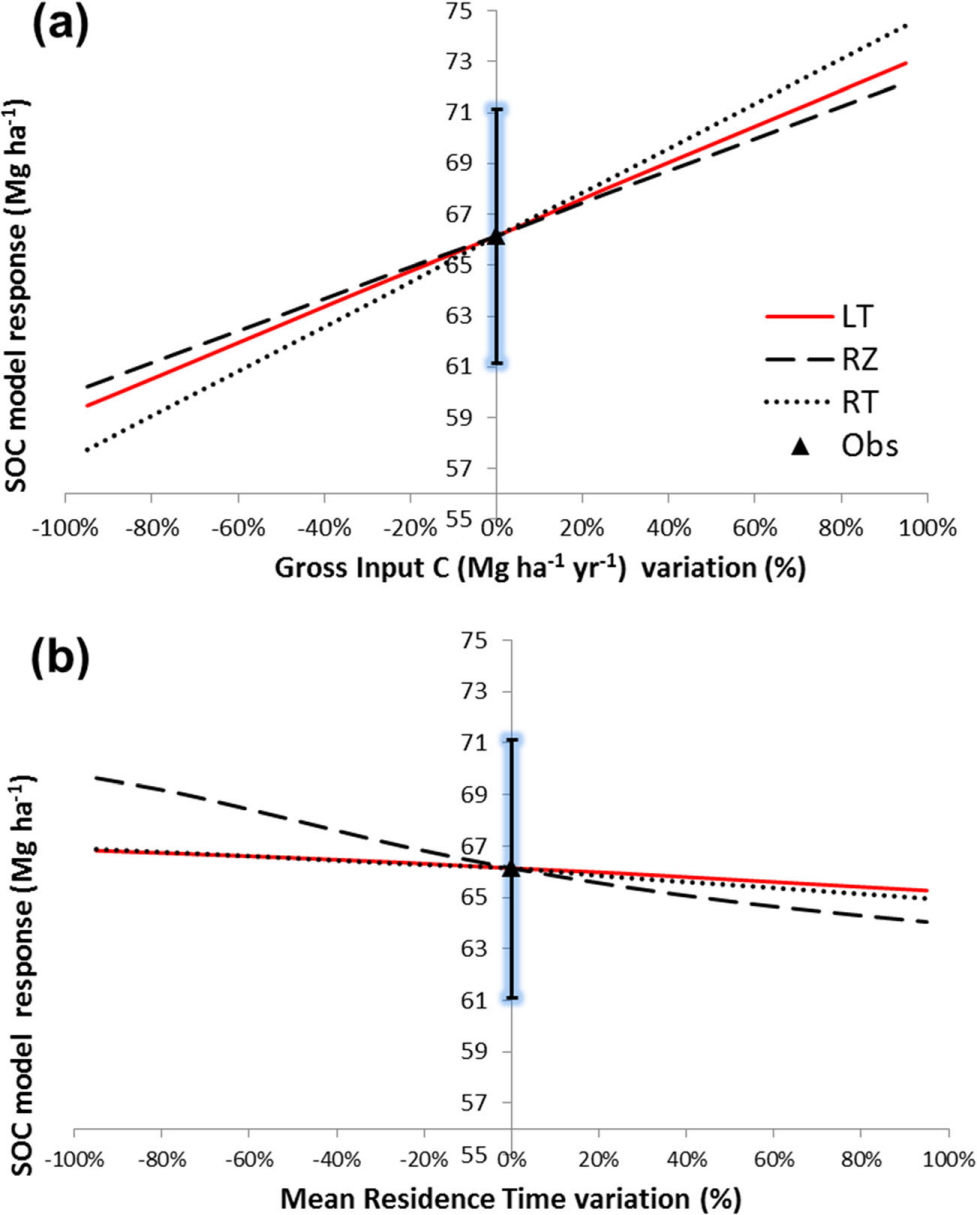
Sensitivity analysis showing the response of soil organic carbon (*SOC*) modelled under a 14-year *Miscanthus* crop using disaggregated inputs in terms of the change from the observed (*Obs*) value of **a** gross input of C and **b** mean residence time (*MRT*) of litter (*LT*), rhizome (*RZ*) and root (*RT*)

Based on model calibration using a 14-year-old *Miscanthus* experiment at Rothamsted (UK), we analysed the sensitivity of the modelled SOC to the input and turnover rates of segregated residues (Fig. 5). The optimised parameter set assumed yearly C gross inputs for litter, root and rhizome of 1.8, 2.2 and 2.3 Mg ha^−1^, respectively, and an MRT for litter/root and rhizomes of 2.3 and 8 years, respectively. The sensitivity analysis showed that for each 1 % change of gross residue C input, the simulated SOC changed by 0.13, 0.11 and 0.1 % for root, litter and rhizome, respectively (Fig. 5a). For a 1 % change of respective residue MRT, the simulated SOC changed by 0.02, 0.01 and 0.05 % for root, litter and rhizome, respectively (Fig. 5b).

Figure 5a clearly implies that not only yield but harvest management (e.g. removal of residues in *Miscanthus*) would affect SOC through the input rate of surface litter which is an important input of ecosystem models [151, 168] and LCA for energy crops [177]. There are very few data for *Miscanthus* [62, 65, 150], switchgrass [178, 179], willow [143, 180] or poplar [22] available. None of these would allow deriving a dose–response function.

Using reviewed parameters for residue deposition and decomposition under SRC willow (Tables 1 and 2), we simulated a very similar ∆SOC as under *Miscanthus*. However, the simulated cumulative CO_2_ emission was 20 % higher than under *Miscanthus*, because the turnover of fine willow roots is 17 times faster than *Miscanthus* roots.

### Further Development Needs

What are the key points that can be derived from the review of the evidence base and its current status for modelling C sequestration (∆SOC) under PECs?

#### There Is No Steady State!

The evidence presented above clearly shows that there is no justification for the assumption of a steady-state sequestration rate as stated by others [8, 136]. The opposite is evident: initial sequestration rates are low due to small inputs during the establishing phase [25, 43, 46, 60, 86, 106, 134], which could depend on the productivity and fertility of the soil, its former land use and planting material/success. Although not significant, initial SOC gains are greater on former arable than on former grassland when planted with *Miscanthus* in spite of greater—probably yield-dependent—sequestration rates on grassland [134]. A long resilience phase for SOC was seen under *Miscanthus* established on former grassland [86], and no change of SOC was measured under 19-year-old willow SRC [143]. A key to this phenomenon could be the high losses of SOC under grass which are likely to be related to higher microbial biomass in the soil of former grassland [181]. Our scenario analysis of a compartmentalised residue input and turnover model shows that differentiation in turnover rates can impact on the dynamics. The modelling of SOC fractionation seems essential for the persistence and sustainability of C sequestration, showing that initially new SOC is part of easily decomposable POM [139] and later part of the inter-aggregate fraction [25, 45]. This would need a series of new experiments to see the decrease of each fraction after returning to arable.

#### Interaction Between Crop Growth and Soil

Root dry matter in the surface horizon is an order of magnitude larger than in the subsoil [31, 32, 80, 84]. This affects the water extraction dynamics and the root exudates. To simulate hydrological effects on the SOC decomposition phenomena, numerous modelling approaches exist. The effect of soil moisture is complex and non-linear; a single moisture threshold for decreased microbial activity and soil respiration does not exist [14]. Usually, process-based SOC models [41, 182] have decomposition reduction functions based on soil moisture, derived from the soil water potential or soil water content. Bauer et al. [41] compared various decomposition routines in a unique modified version of RothC and showed 2 % sensitivity of CO_2_ emissions due to soil moisture variation. The uncertainty of soil moisture derived from pedotransfer functions can affect C flux simulations [183]. Bulk density in particular is a dynamic property when changing from arable to perennial agricultural systems [24], and its variation increases the uncertainty of stock changes [80, 164, 184] and needs to be accounted for to capture the effect of spatial heterogeneity on SOC dynamics and sequestration.

#### Scaling Up—Is There Enough Knowledge?

Empirical coefficients, such as those used in the IPCC Tier 1 method [39], are insufficient to account for SOC sequestration under PECs. Spatial variation accounted for by GIS-based simulation provides yield-dependent potential C inputs and sequestration in soils [63]. Their assumptions that the soil-crop system was at potential productivity, and equilibrium could be wrong where net SOC formation is delayed [86], cover is patchy [46, 139] or residues vary in their decomposability [108]. There is no doubt that, technically, various plant ecosystems and a SOC decomposition model of desired complexity can be integrated with some empirical method for assessing site/soil-specific SOC stock changes, as shown for the GEFSOC system [39]. Currently, the experimental evidence for the effect of soil texture and baseline SOC on sequestration potential [63] is so small that a general relationship for its spatial extrapolation is not credible. Data for ∆SOC from marginal soils with a low baseline SOC, such as the site in Ireland [135], are target areas but are not available to validate a generalised model. Indeed, topography and soil distribution maps can be included as inputs to process-based models to simulate the impacts on growth and SOC for PECs.

An alternative up-scaling technique is to run C process models (i.e. RothC) at small scales to initialise larger spatial regression models [185]. However, the limitations of process-based models become more apparent when simulating at larger scales with regard to their limits to consider the effect of soil depth [168], adequate temporal resolution [171] and surface litter decomposition [173, 174]. It is difficult to obtain sufficiently detailed and widespread data to generalise and verify a generic process description.

## Concluding Remarks

Is the jury still out? What are the certainties and uncertainties with regard to the assessment of carbon sequestration under PECs? We draw attention to four major points when considering this:

- First, our review shows annual ∆SOC of HCG and SRWC exceeding the minimum sequestration rate, and more certainty applies to C4-HCG than C3-SRWC due to isotope (^13^C) use. Based on the C inputs retained, ∆SOC (Fig. 4), one can distinguish retentive HCG (30–55 %) from transformative SRWC (19–33 %) systems.
- Second, underpinning evidence for the C balance components is more certain for AGB than BGB inputs: (a) litter fall and harvest residues are more easily measured than roots and rhizomes/stool and (b) gross inputs of BGB components profoundly depend on their MRT. Turnover is derived from time series estimating productivity/mortality over standing biomass, but time series are the exception over single ‘snapshot’ observations.
- Third, severe restrictions apply to the validity of current *in situ* residue turnover rates due to technical limitations (litter bags) and steady-state assumptions. Laborious alternative methods have filled knowledge gaps but described dynamics in young plantations, which may not be extrapolated to mature stands. C sequestration implies a long-term view and analysis on SOC fractions that represent a turnover time in excess of 25 to 50 years.
- Fourth, effects from the environment, fertility and management, e.g. harvest intensity of the systems, are not covered in dose–response experiments, and evidence for target ecosystems, like marginal land, is underrepresented.

In terms of modelling, reliable projections can only be achieved if simulations capture short- and long-term dynamics, synthesising data from chrono-sequences, disaggregating inputs and understanding C fluxes within SOC fractions. Experimental evidence for the SOC dynamics, size and persistence of the SOC pools prior to establishment, during the lifetime and after termination of the PECs must underpin modelling.

## Glossary

AGB: Aboveground biomass
BGB: Belowground biomass
Component: Pool or residue fraction of A/BGB within a systems model
Decomposition/turnover rate (*k*): Portion of [stored or added] C lost from a given pool (time, e.g. year^−1^)
DOC: Dissolved organic C
FACE: Free air C enrichment
GHG: Greenhouse gas
MRT: Mean residence time, average storage time [years] for a given residue or C pool; 1/*k* assuming zero order
Half-life: Time in which 50% of the C component is degraded; assuming first-order decay: *t*_1/2_ = ln2 / *k*
Soil C retention or storage: C retained without specifying a defined time span
Soil C sequestration: C ‘sequestered’ when stored in soil for at least 25 years
HCG: Herbaceous crops and grasses
PEC: Perennial energy crops
SRC: Short-rotation coppice
SRF: Short-rotation forestry
SRWC: Short-rotation woody crops (SRC and SRF)
LCA: Life cycle assessment
(i)LUC: (Indirect) land use change
